# Network Analysis of Induced Neural Plasticity Post-Acceptance and Commitment Therapy for Chronic Pain

**DOI:** 10.3390/brainsci11010010

**Published:** 2020-12-23

**Authors:** Sarah K. Meier, Kimberly L. Ray, Noah C. Waller, Barry C. Gendron, Semra A. Aytur, Donald A. Robin

**Affiliations:** 1 Department of Communication Sciences and Disorders, University of New Hampshire, Durham, NH 03824, USA; ncw1004@wildcats.unh.edu (N.C.W.); don.robin@unh.edu (D.A.R.); 2 Department of Psychology, University of Texas, Austin, TX 78712, USA; kimray@utexas.edu; 3 Seacoast Area Physiatry, Portsmouth, NH 03801, USA; barry.gendron@wdhospital.org; 4 Department of Health Management and Policy, University of New Hampshire, Durham, NH 03824, USA; semra.aytur@unh.edu; 5 Interdisciplinary Program in Neuroscience and Behavior, University of New Hampshire, Durham, NH 03824, USA; 6 Department of Biological Sciences, University of New Hampshire, Durham, NH 03824, USA

**Keywords:** acceptance and commitment therapy, chronic pain, fMRI, functional connectivity, graph theory

## Abstract

Chronic musculoskeletal pain is a costly and prevalent condition that affects the lives of over 50 million individuals in the United States. Chronic pain leads to functional brain changes in those suffering from the condition. Not only does the primary pain network transform as the condition changes from acute to persistent pain, a state of hyper-connectivity also exists between the default mode, frontoparietal, and salience networks. Graph theory analysis has recently been used to investigate treatment-driven brain network changes. For example, current research suggests that Acceptance and Commitment Therapy (ACT) may reduce the chronic pain associated hyper-connectivity between the default mode, frontoparietal, and salience networks, as well as within the salience network. This study extended previous work by examining the associations between the three networks above and a meta-analytically derived pain network. Results indicate decreased connectivity within the pain network (including left putamen, right insula, left insula, and right thalamus) in addition to triple network connectivity changes after the four-week Acceptance and Commitment Therapy intervention.

## 1. Introduction

Chronic musculoskeletal pain (CMP) affects the lives of over 50 million individuals in the US at a rate of 11–40% with contributions to medical costs and intervention programs being over $550 billion each year [1]. CMP manifests after an eventual transition from acute pain and central cognitive processes largely explain the persistent nature of pain in the instance of musculoskeletal damage [2]. Co-morbid cognitive changes associated with chronic pain are often modulated by emotions [3]. These cognitive and emotional changes involve mood, overall psychological state, meaning-related thoughts, and the ability to learn new information. Thus, CMP commonly occurs concomitantly with depression, anxiety, fatigue, difficulty remembering, and difficulty concentrating. These coexisting conditions can result in substantial disabilities or impairment in work participation, social interactions, and self-care practices [4]. These co-morbid effects are associated with functional changes in specific brain circuitry and appear to alter the perception of and self-reflection about the pain itself [5].

Brain changes in CMP are widespread and involve the pain network as well as sensory, emotional, and cognitive control networks that process information [6]. Areas commonly included in the pain network are the thalamus, the insular cortex, the primary and secondary somatosensory cortices, the anterior cingulate cortex, and the prefrontal cortex. It has been shown that areas of the pain network that deal with emotional and motivational modulations are active during pain [6]. This pain network, a sensorimotor network, is functionally connected to higher cognitive networks that include the default mode (DMN), frontoparietal (FPN), and salience (SN) networks. All of these networks are altered in CMP.

Previous work has shown that chronic pain conditions impact the brain via networks noted above [7]. The DMN is a network noted to be more active when an individual is not participating in any given mental task [8,9]. Increased posterior DMN connectivity (involving insular and anterior cingulate regions) proportional to the intensity of pain has previously been reported [10]. The FPN is typically featured in cognitive control aspects of neural processing. Data show that the FPN predicts how pain intensity will progress for those experiencing chronic pain particularly within the first three months of the condition [11]. The SN is a prominent network in emotional control and social behavior especially regarding detection of salient stimuli. The SN can also become active after painful stimuli causing attention to shift away from the point of focus [12]. Hemington and colleagues also state that the SN abnormalities have been correlated with chronic pain, continuous pain stimuli, and pain-related symptomology.

Not only does each network independently demonstrate changes in functional connectivity in response to different types of chronic pain stimuli, it has been demonstrated that there is elevated functional connectivity among the DMN, FPN, and SN in individuals suffering from CMP [12,13,14,15,16,17,18]. Functional connectivity measures the temporal connection between distant neurophysiological brain signals. These three core networks, referred to as the “triple network,” have become crucial in understanding higher levels of cognitive functioning and reasons it may become abnormal [19,20].

The current investigation builds upon Aytur and colleagues’ work (under review, [21]) on behavioral changes and neurological changes in connectivity within and between the DMN, FPN, and SN as a result of four weeks of Acceptance and Commitment Therapy (ACT) for chronic pain. Hayes and colleagues describe ACT as a “third generation approach” that is particularly sensitive to psychological phenomena and emphasizes contextual changes and directly targeted approaches to create a broad and flexible range of mental capabilities [22]. It is considered a “well-established” treatment for CMP by the American Psychological Association. This therapy focuses on (1) observing normally occurring negative thoughts and feelings as they arise in one’s mind without trying to alter them in any way and (2) regularly behaving in accordance with personal values and life goals [23]. The acceptance technique of ACT has been shown to produce lower self-rated pain intensity, depression, pain-related anxiety, and pain avoidance while increasing physical, social, and occupational ability [23]. It is believed that, through the mindfulness and acceptance techniques of ACT, psychological flexibility (an individual’s ability to continue participating in value-based behaviors during aversive experiences) increases while reducing responses to chronic pain sensations. These outcomes indicate a strong rationale for attempting the ACT practice with a CMP population.

Examining resting state network connectivity (in the DMN, FPN, SN, and pain network) of individuals with CMP who were exposed to an ACT intervention can provide insight into the neural substrates underlying the chronic pain experience. The Network-Based Statistic (NBS) [24] is the focus in the following evaluation of neurological changes pre- and post-ACT. The concept of graph theory is applicable here as within-subject network comparison may reveal connectivity abnormalities. These abnormalities may be related to their CMP condition or the neurological changes induced post-ACT. Graph theory, namely the NBS, can be used to examine connectivity across regions of the brain, the integration of information between brain networks, and the roles that certain brain regions may play in communication between themselves [25]. The application of graph theoretical analysis to the functional brain allows us to model the brain as a “network of networks.” Within these networks are “nodes,” representing brain regions, and “edges,” representing functional connections between any two nodes or brain regions [26,27,28]. An advantage of this type of network analysis is that it allows us to examine the relationship of multiple brain networks with each other, something other MRI approaches do not allow for. Additionally, this analysis allows for examination of changes across various time points.

Prior to this study, as well as Aytur and colleagues’ study [21], little was known about how the brain can change in response to specific interventions in certain disease states, such as CMP. The extent to which ACT interacts with the hypothesized neural systems underlying chronic pain was also unknown. The present study further investigated relationships between the default mode, frontoparietal, and salience networks. Specifically, this work extends the investigation of the triple network by also examining the role of a pain network (driven by a recent meta-analysis) in relation to CMP. While the triple network previously showed reduced connectivity, it is unknown how that affects potential change in the pain network. This work is important as subjective reports suggest decreased levels of perceived pain due to ACT, a treatment focusing on cognition and emotion rather than pain itself. As such, the primary hypothesis is that connectivity of the pain network should not change. However, connectivity between the triple network and the pain network may change post-ACT since pain itself affects cognitive and emotional brain networks.

## 2. Materials and Methods

Nine female participants (age 47.59 years ± 16.54 years; eight right-handed) completed the entire four-week ACT protocol of Aytur and colleagues’ study [21] after being recruited through community-based health care clinics and providing their informed consent. The study (#6991) was approved by the University of New Hampshire Institutional Review Board on July 19, 2018. The nine participants were referred from an outpatient physician certified in Physical Medicine and Rehabilitation with Subspecialty Certification in Pain Medicine. The practice involves management of both acute pain and CMP, with a higher prevalence of females versus males with CMP. Although both males and females were referred for study participation, the study protocol required daytime commitment and out-of-state travel that precluded participation by both male and female subjects that were referred for participation. Participants were required to be at least 18 years of age, English-speaking, and to have had CMP for at least three months. Participants had to have an average pain interference score of at least four on question nine of the Brief Pain Inventory [29]. Ten participants began initial testing. However, one individual ended the baseline scan early due to a medical condition, was eliminated from the study at that time, and was prorated for their time in the study.

Participants completed a series of neuropsychological assessments in their pre-treatment session to determine baseline measures of cognitive ability, quality of life, and pain level. Select domains from the NIH Cognition Battery, PROMIS (Patient-Reported Outcomes Measurement Information System; regarding pain interference, pain intensity, etc.), and Neuro-QoL^TM^ (Quality of Life in Neurological Disorders; regarding sleep, depression, anxiety, etc.) were obtained from the NIH Toolbox and administered via iPad. Any additional assessments were administered via paper copy. The full set of assessments (Supplementary Materials, Table S1) was administered to each subject both pre-and post-ACT. Analysis methods and results for behavioral data are in Supplementary Materials.

Resting-state functional magnetic resonance imaging (rsfMRI) data were collected during two separate scan sessions using a Siemens Three Tesla (3T) Magnetom Prisma scanner at Boston University’s Cognitive Neuroimaging Center in Boston, MA, USA. The baseline MRI scan was collected before treatment and a second MRI scan was collected within two weeks post-treatment. For each of these MRI visits, structural MP-RAGE images were collected (TR/TE = 2.53 s/1.32 ms, flip angle = 7°, field of view (FOV) = 256 × 320 mm, 0.8 mm^3^ resolution), followed by two eight-minute scans to obtain resting state functional images using a T2* weighted Echo Planar Imaging (EPI) sequence (2.5 mm^3^ resolution, 60 slices, TR/TE = 1.2 s/30 ms, 300 volumes, FOV = 205 mm, multi-slice interleaved ascending). All nine participants underwent this imaging protocol and were instructed to lie still in the scanner with eyes open, fixating on the crosshair placed in their field of view. Each participant completed an MRI safety screening prior to scanning to rule out any medical or other issues that may have jeopardized their safety or excluded them from the study.

The entire implementation of ACT consisted of eight 90-min twice weekly group sessions administered over four continuous weeks. The first four-week protocol included four of the female participants (ages 38–66) and the second four-week protocol included five of the female participants (ages 20–60). Each session was set up to consist of seven steps (see Supplementary Materials, Figures S1 and S2). These two four-week ACT sessions were administered by two licensed recreational therapists who received training from an ACT specialist (Potter, J.).

All imaging data collected from the MRI scanner were preprocessed using standard approaches in Statistical Parametric Mapping software, version 12 (SPM12) [30], implemented via MATLAB 9.3 (R2017b) [31]. Functional data were realigned and co-registered to the standard Montreal Neuroimaging Institute (MNI) template, motion corrected, slice-timing corrected, normalized to remove any individual variability for between subject comparisons, and smoothed to increase signal to noise ratio, all in SPM12. Corrections for head movement were applied including smoothing using a 3-mm full-width at half maximum Gaussian kernel and a band-pass filter, which preserved frequencies between 0.01 and 0.08 Hz.

**Figure 1:**
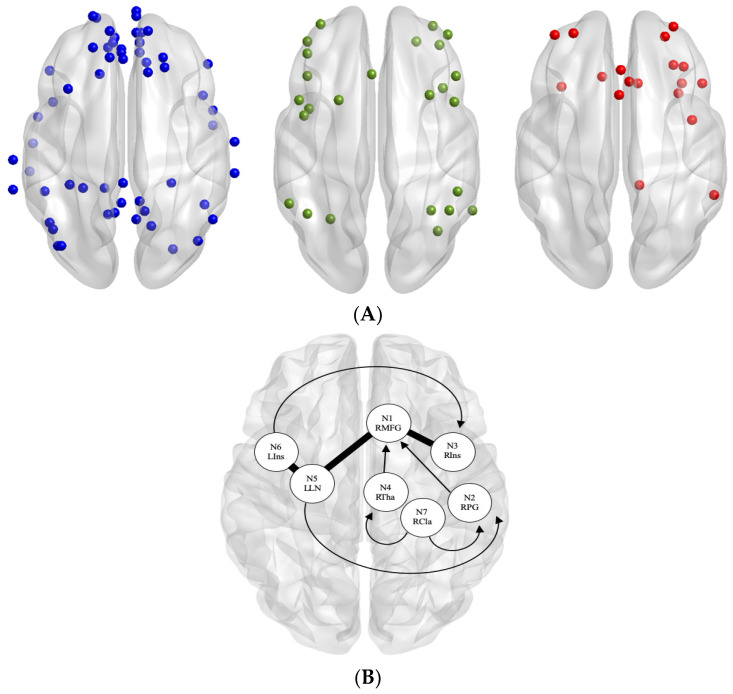
(**A**) Original nodes used in each network: (**blue**) default mode network (DMN), (**green**) frontoparietal network (FPN), and (**red**) salience network (SN). (**B**) Pain nodes used, derived from chronic pain functional meta-analytic connectivity modelling (MACM) results [35]. Regions of interest (ROI) labels: N1 RMFG = right medial frontal gyrus, N2 RPG = right postcentral gyrus, N3 RIns = right insula, N4 RTha = right thalamus, N5 LLN = left lentiform nucleus, N6 LIns = left insula, and N7 RCla = right claustrum. Arrows denote one-way connections projecting from the starting node. Thick non-arrows denote two-way connections between nodes.

Each participant’s brain data were parcellated into specific regions of interest (ROIs) for the DMN, FPN, and SN using a Multi-image Analysis GUI (Mango) [32] selected from the Power atlas [33]. The mean time course (BOLD signal changes across time) within these seed regions was extracted from the residual images collected before and after ACT using Response Exploration for Neuroimaging Datasets (REX) [34]. Functional connectivity estimates across all selected ROIs were then calculated using the pairwise Pearson correlation of the region’s time course. Connectivity matrices from pre-ACT and post-ACT for all participants were reduced to 101 × 101 ROIs for the combination of DMN, FPN, and SN (Figure 1A; see Supplementary Materials, Table S2 for *x, y, z* coordinates of nodes in each of the four networks).

The pain matrix was created from a meta-analysis of regional activation in chronic pain patients [35]. We chose to include functionally and meta-analytically derived nodes to be comparable to the functionally and meta-analytically derived Power atlas nodes. Waller and colleagues used the activation likelihood estimate (ALE) [36] to identify brain regions consistently activated during pain induction (for 419 subjects and 398 coordinate foci) and then applied meta-analytic connectivity modeling (MACM) [37] to yield co-activation patterns between those regions. This meta-analysis of chronic pain identified seven brain areas that showed increased activation during pain induction compared to a control condition (Figure 1B; right medial frontal gyrus, bilateral insula, right postcentral gyrus, left lentiform nucleus, right thalamus, and right claustrum). We refer to these seven brain regions as the pain network, which were included in our analyses as a fourth network of interest for subsequent graph analyses.

The NBS [24] was used to identify and evaluate the extent of functional connectivity changes in the DMN, FPN, SN, and pain network between pre-ACT and post-ACT. The NBS calculates the difference between the pre-ACT and post-ACT functional connectivity matrices across all participants. This graph theory-based method utilizes a general linear model-based approach to identifying functional connectivity changes within a graph. An advantage of the NBS is that it controls the family wise error rate when mass univariate testing is performed at every connection comprising the graph. Results presented represent network connectivity differences for *p* > 0.05 at 10,000 permutations. Explicitly, permutation testing is used to ascribe a *p*-value controlled for the family wise error to each connected component based on its size [24]. While we predicted that we would observe decreases in connectivity as a result of ACT, NBS *t*-tests are one-tailed; therefore, we tested for both increases and decreases in connectivity between pre- and post-ACT across a range of *t*-statistic thresholds to ensure that reported significant findings are robust.

In our initial report [21] we tested for changes in brain networks underlying ACT-related outcomes in those with CMP using only triple network nodes without the inclusion of pain nodes (*t* > 2.1 for the 101 nodes of the DMN, FPN, and SN). We observed effects in 10 nodes out of 101 triple network nodes and used those same 10 for the following analysis in conjunction with Waller and colleagues’ [35] pain matrix of seven nodes found via ALE (17 nodes in total). This takes into account whether ACT induced changes may be present between the pain network and the three cognitive networks. Finally, we tested for more widespread network effects in a follow up NBS analysis that included the original (101) triple network nodes in addition to Waller and colleagues’ seven pain nodes (108 nodes in total).

## 3. Results

The present study investigated functional relationships between the default mode, frontoparietal, and salience networks by also examining the role of a pain network (driven by a recent meta-analysis) in relation to CMP. Through NBS, a range of *t*-statistic thresholds were tested to identify significant functional connectivity differences across the four networks of interest.

### rs-fMRI Connectivity Effects

**Figure 2:**
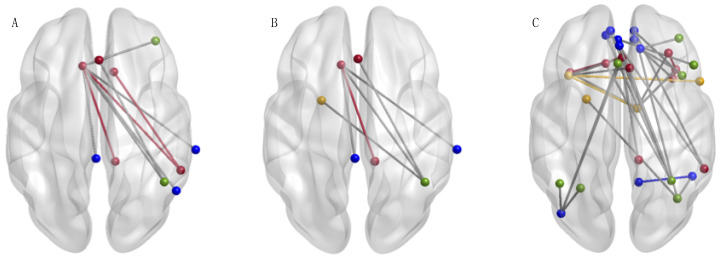
The Network-Based Statistic (NBS) [24] identified resting state functional connectivity effects of Acceptance and Commitment Therapy (ACT) between DMN (blue), FPN (green), SN (red), and the pain network (yellow). This shows decreased functional connectivity (post-ACT > pre-ACT) for (**A**) 10 nodes connected by 10 edges (*t* > 2.5; *p* = 0.05, 10,000 permutations), (**B**) 7 nodes connected by 6 edges (*t* > 2.5; *p* = 0.004, 10,000 permutations), and (**C**) 31 nodes connected by 34 edges (*t* > 3.4; *p* = 0.036, 10,000 permutations). Within network connectivity changes were represented by functional connections of color (a connection within SN nodes (red) will be represented by a red edge, etc.). See Supplementary Materials (Table S3) for details of which nodes of each network were included in these significant networks. Figures were created using BrainNet [38].

Of the 101 nodes in the DMN, FPN, and SN we examined, the NBS identified only a subset of 10 nodes connected by 10 edges that exhibited effects of ACT. This collection of 10 nodes and 10 edges showed reduced connectivity from pre-ACT to post-ACT (Figure 2A; *t* > 2.5 *p* = 0.05). This network is representative of the same finding in our previous report [21]. We subsequently tested for effects of ACT on the 10 nodes identified in the previous analysis (Figure 2A) combined with the seven pain nodes (17 nodes total). In doing so, the NBS revealed lowered levels of connectivity as a result of ACT across seven nodes and six edges corresponding to DMN, FPN, SN, or the pain network when aggregated together (Figure 2B; *t* > 2.5 *p* = 0.004). Our final analysis examined effects of ACT across all nodes in the four networks of interest, which included the original 101 triple network nodes and the seven pain nodes (108 nodes total). Effects of ACT were demonstrated by lowered levels of connectivity from pre- to post-ACT across 31 nodes and 34 edges corresponding to DMN, FPN, SN, or the pain network when aggregated together (Figure 2C; *t* > 3.4 *p* = 0.036). Nodes of the pain network that were involved in decreased functional connections include the left putamen, right insula, left insula, and right thalamus. The presented network effects were significant across multiple *t*-statistic threshold. No increases in connectivity were observed in any NBS test that would correspond to pre-ACT > post-ACT.

## 4. Discussion

The current study investigated the extent to which Acceptance and Commitment Therapy induced network-connectivity changes in persons suffering from chronic pain. Previous studies have identified neurophysiological changes in the instance of chronic pain in the thalamus, insular cortex, anterior cingulate cortex, and prefrontal cortex, as well as the primary somatosensory cortex and parietal cortex [6,39]. Such neural mechanisms have been linked to catastrophizing one’s pain [40]. Negative patterns form when brain changes occur in these regions. This leads to thoughts of helplessness, pessimism, rumination, and a perceived poor quality of life [40]. Supplemental information and current research [21] describe strong, positive behavioral changes after ACT intervention for participants.

Previous studies also present the potential for alterations of the DMN [10,16,41,42,43,44], FPN [11,45,46,47], and SN [12,48,49] in those experiencing chronic pain, exposing the necessity to further investigate neurological mechanisms underlying chronic pain. Hypotheses of Aytur and colleagues’ study [21] were rooted in the theory that hyper-connectivity between these three networks (DMN, FPN, and SN) exists in the brains of individuals with chronic pain [12,16,17]. Those results suggest that levels of heightened connectivity between the triple network returned to a lower, healthier level of connectivity post-ACT. The current study utilized this information to hypothesize that the cause may be alterations to pain-related cognitive and emotional networks and consequently examined relationships involving the pain network. Data demonstrating decreased functional connectivity involving pain network nodes support this hypothesis.

The Network-Based Statistic [24] was examined to determine functional connectivity changes within and between specific networks. Through examining the significant triple network (10 nodes from initial investigation, [21]) in addition to the pain network of seven nodes (17 nodes total; see Figure 2B), involvement of the pain network was revealed. Subsequently, expanding the analysis to encompass the triple network and the pain network (108 nodes total; see Figure 2C) revealed decreases pre- to post-ACT of pain nodes as well as each of the triple networks. Four pain nodes displayed functional connectivity changes associated with ACT treatment. This unexpected finding suggests that connectivity between both the left putamen and right insula, as well as the left insula and right thalamus, of the pain network decreases pre- to post-ACT. Activation of these regions has been linked to thoughts of helplessness, pessimism, and rumination in the instance of CMP [40]. So, decreased functional connectivity in these regions may contribute to enhanced quality of life that is reported post-ACT [21,40].

Changes in cognitive and emotional networks indicate the participants’ ability to deal with pain more efficiently after ACT intervention. Despite prior research suggesting that ACT can reduce pain symptomatology without altering the pain network, the current analyses do expose changes to the pain network as a result of ACT. This may be due to the use of ALE [36] to define regions activated during chronic pain processing. Other (non-meta-analytically derived) nodal assignments may not be as well suited for chronic pain conditions specifically. These functional connectivity changes involving regions of the DMN, FPN, SN, and pain network (alongside previously reported positive behavioral outcomes, [21]) are convincing underlying reasons for the way ACT effectively alleviates chronic pain symptomology.

We note potential limitations within the study. A main limitation in the current study is the sample and its size. This subject group had variability within their individual pain experiences (fibromyalgia, genetic conditions, etc.) as well as their age. The sample was limited to white female participants. Selection bias may play a role such that participants who self-selected to participate may have been different from the general population. Although this study reviews neural pathways in ACT intervention in females, additional study among participants of all genders should be considered. Another limitation lies within the lack of a control group. No comparisons were made between the individuals with chronic pain and a healthy control population who were not experiencing chronic pain. Lastly, the long-term effects of ACT on pain management are not well documented. Thus, additional follow up data (a retention time point) would provide much needed information on the state of neural changes resulting from ACT. It is possible that further normalization of brain connectivity could occur especially if the study participants continue utilizing the mindfulness techniques learned over the course of the treatment. These factors should be addressed as the investigation of the relationship between chronic pain, functional connectivity, and ACT grows.

## 5. Conclusions

We examined the mechanism of action underlying an evidence-based treatment approach for chronic pain using novel graph theory analysis. Our findings add to results of prior research suggesting that Acceptance and Commitment Therapy normalizes the hyper-connectivity that exists between the default mode, frontoparietal, and salience networks of individuals with chronic pain. This work also suggests that connectivity involving pain nodes is also alleviated. The current study contributes to the necessary knowledge of neurological mechanisms underlying chronic pain conditions as a means of optimizing the ACT treatment. Analyses of alternative treatments for CMP are important as traditional pain medications do little to affect both the hyper-connectivity and dully perceived quality of life existing in those with chronic pain. Most common medications (opiates, muscle relaxants, anti-depressants, e.g.,) aim to reduce or manage the actual pain sensation but can often lead to unwanted side effects and major life changes [23]. Non-pharmacologic treatments, such as ACT, need to continue to be considered as part of a multi-modal toolbox for pain management. Based on clear guidance from chronic pain experts, including those from the Institute of Medicine (IOM), clinician and patient understanding are paramount in treating and preventing the worsening of CMP. This study improves the mechanistic understanding of ACT, aligning with the IOM’s CMP treatment goal of provider and patient education.

## Supplementary Materials

The following are available online at https://www.mdpi.com/2076-3425/11/1/10/s1, Figure S1: The seven-step timeline of each individual 90-min ACT session, Figure S2: A basic timeline of the entire study, Table S1: List of behavioral and neuropsychological assessments, Table S2: List of all *x*, *y*, *z* coordinates for the nodes involved in each of the four networks, Table S3: List of the nodes presented in Figure 2A,B,C, Table S4: List of significant edges of the salience network involving four nodes, Table S5: List of the significant edges of triple network (t > 3.4), Table S6: List of significant edges of the triple network (t > 2.1), Table S7: Statistically significant changes scores of behavioral assessments administered, Table S8: Correlations between significant assessment and edge scores.

## References

[1] Dahlhamer J., Lucas J., Zelaya C., Nahin R., Mackey S., DeBar L., Helmick C. (2018). Prevalence of chronic pain and high-impact chronic pain among adults—United States, 2016. Morb. Mortal. Wkly. Rep..

[2] Mano H., Kotecha G., Leibnitz K., Matsubara T., Sprenger C., Nakae A., Seymour B. (2018). Classification and characterisation of brain network changes in chronic back pain: A multicenter study. Wellcome Open Res..

[3] Reddan M.C., Wager T.D. (2018). Modeling pain using fMRI: From regions to biomarkers. Neurosci. Bull..

[4] Pitcher M.H., Von Korff M., Bushnell M.C., Porter L. (2019). Prevalence and profile of high-impact chronic pain in the United States. J. Pain.

[5] Bushnell M.C., Čeko M., Low L.A. (2013). Cognitive and emotional control of pain and its disruption in chronic pain. Nat. Rev. Neurosci..

[6] Morton D., Jones A., Sandhu J. (2016). Brain imaging of pain: State of the art. J. Pain Res..

[7] Mitsi V., Zachariou V. (2016). Modulation of pain, nociception, and analgesia by the brain reward center. Neuroscience.

[8] Mak L.E., Minuzzi L., MacQueen G., Hall G., Kennedy S.H., Miley R. (2017). The default mode network in healthy individuals: A systematic review and meta-analysis. Brain Connect..

[9] Raichle M.E. (2015). The brain’s default mode network. Annu. Rev. Neurosci..

[10] Kuner R., Flor H. (2017). Structural plasticity and reorganisation in chronic pain. Nat. Rev. Neurosci..

[11] Pfannmöller J., Lotze M. (2019). Review on biomarkers in the resting-state networks of chronic pain patients. Brain Cognit..

[12] Hemington K.S., Wu Q., Kucyi A., Inman R.D., Davis K.D. (2016). Abnormal cross-network functional connectivity in chronic pain and its association with clinical symptoms. Brain Struct. Funct..

[13] Cauda F., Palermo S., Costa T., Torta R., Duca S., Vercelli U., Torta D.M.E. (2014). Gray matter alterations in chronic pain: A network-oriented meta-analytic approach. NeuroImage Clin..

[14] Cottam W.J., Iwabuchi S.J., Drabek M.M., Reckziegel D., Auer D.P. (2018). Altered connectivity of the right anterior insula drives the pain connectome changes in chronic knee osteoarthritis. Pain.

[15] Doll A., Hölzel B.K., Boucard C.C., Wohlschläger A.M., Sorg C. (2015). Mindfulness is associated with intrinsic functional connectivity between default mode and salience networks. Front. Hum. Neurosci..

[16] Napadow V., LaCount L., Park K., As-Sanie S., Clauw D.J., Harris R.E. (2010). Intrinsic brain connectivity in fibromyalgia is associated with chronic pain intensity. Arthritis Rheum..

[17] Van Ettinger-Veenstra H., Lundberg P., Alföldi P., Södermark M., Graven-Nielsen T., Sjörs A., Gerdle B. (2019). Chronic widespread pain patients show disrupted cortical connectivity in default mode and salience networks, modulated by pain sensitivity. J. Pain Res..

[18] Zhao Z., Huang T., Tang C., Ni K., Pan X., Yan C., Luo Y. (2017). Altered resting-state intra- and inter- network functional connectivity in patients with persistent somatoform pain disorder. PLoS ONE.

[19] Menon V. (2011). Large-scale brain networks and psychopathology: A unifying triple network model. Trends Cognit. Sci..

[20] Menon V. (2018). The Triple Network Model, Insight, and Large-Scale Brain Organization in Autism. Biol. Psychiatry.

[21] Aytur S.A., Ray K.L., Meier S.K., Campbell J., Gendron B., Robin D.A. (2020). Neural mechanisms of acceptance and commitment therapy for chronic pain: A network-based fMRI approach. MedRxiv.

[22] Hayes S.C., Luoma J.B., Bond F.W., Masuda A., Lillis J. (2006). Acceptance and Commitment Therapy: Model, processes and outcomes. Behav. Res. Ther..

[23] Dahl J., Lundgren T., Baer R.A. (2006). Acceptance and commitment therapy in the treatment of chronic pain. Mindfulness-Based Treatment Approaches: Clinician’s Guide to Evidence Base and Applications.

[24] Zalesky A., Fornito A., Bullmore E.T. (2010). Network-based statistic: Identifying differences in brain networks. NeuroImage.

[25] Smith S.M., Vidaurre D., Beckmann C.F., Glasser M.F., Jenkinson M., Miller K.L., Van Essen D.C. (2013). Functional connectomics from resting-state fMRI. Trends Cognit. Sci..

[26] Sporns O. (2018). Graph theory methods: Applications in brain networks. Dialog. Clin. Neurosci..

[27] Farahani F.V., Karwowski W., Lighthall N.R. (2019). Application of graph theory for identifying connectivity patterns in human brain networks: A systematic review. Front. Neurosci..

[28] Wang J., Ren Y., Hu X., Nguyen V.T., Guo L., Han J., Guo C.C. (2017). Test-retest reliability of functional connectivity networks during naturalistic fMRI paradigms. Hum. Brain Mapp..

[29] Cleeland C.S., Ryan K.M. (1994). Pain assessment: Global use of the Brief Pain Inventory. Annals.

[30] Penny W., Friston K., Ashburner J., Kiebel S., Nichols T. (2006). Statistical Parametric Mapping: The Analysis of Functional Brain Images.

[31] (2010). MATLAB, version 9.9(R2017b).

[32] Kochunov P., Lancaster J., Thompson P., Toga A.W., Brewer P., Hardies J., Fox P. (2002). An optimized individual target brain in the Talairach coordinate system. NeuroImage.

[33] Power J.D., Cohen A.L., Nelson S.M., Wig G.S., Barnes K.A., Church J.A., Petersen S.E. (2011). Functional network organization of the human brain. Neuron.

[34] Duff E.P., Cunnington R., Egan G.F. (2011). REX: Response exploration for neuroimaging datasets. Neuroinformatics.

[35] Waller N.C., Ray K.L., Meier S.K., Aytur S.A., Robin D.A. Regional brain activation in chronic pain: A functional connectivity meta-analysis with healthy controls and chronic pain patients.

[36] Eickhoff S.B., Bzdok D., Laird A.R., Kurth F., Fox P.T. (2012). Activation likelihood estimation revisited. NeuroImage.

[37] Robinson J.L., Laird A.R., Glahn D.C., Lovallo W.R., Fox P.T. (2010). Meta-analytic connectivity modeling: Delineating the functional connectivity of the human amygdala. Hum. Brain Mapp..

[38] Xia M., Wang J., He Y. (2013). BrainNet Viewer: A network visualization tool for human brain connectomics. PLoS ONE.

[39] Seminowicz D.A., Wideman T.H., Naso L., Hatami-Khoroushahi Z., Fallatah S., Ware M.A., Stone L.S. (2011). Effective treatment of chronic low back pain in humans reverses abnormal brain anatomy and function. J. Neurosci..

[40] Zeidan F., Martucci K.T., Kraft R.A., Gordon N.S., McHaffie J.G., Coghill R.C. (2011). Brain mechanisms supporting the modulation of pain by mindfulness meditation. J. Neurosci..

[41] Karafin M.S., Chen G., Wandersee N.J., Brandow A.M., Hurley R.W., Simpson P., Field J.J. (2019). Chronic pain in adults with sickle cell disease is associated with alterations in functional connectivity of the brain. PLoS ONE.

[42] Kornelsen J., Sboto-Frankenstein U., McIver T., Gervai P., Wacnik P., Berrington N., Tomanek B. (2013). Default mode network functional connectivity altered in failed Back surgery syndrome. J. Pain.

[43] Wakaizumi K., Jabakhanji R., Ihara N., Kosugi S., Terasawa Y., Morisaki H., Baliki M.N. (2019). Altered functional connectivity associated with time discounting in chronic pain. Sci. Rep..

[44] Zhang Y., Mao Z., Pan L., Ling Z., Liu X., Zhang J., Yu X. (2019). Frequency-specific alterations in cortical rhythms and functional connectivity in trigeminal neuralgia. Brain Imaging Behav..

[45] Kutch J.J., Labus J.S., Harris R.E., Martucci K.T., Farmer M.A., Fenske S., Mayer E.A. (2017). Resting-state functional connectivity predicts longitudinal pain symptom change in urologic chronic pelvic pain syndrome: A MAPP network study. Pain.

[46] Zheng W., Woo C.-W., Yao Z., Goldstein P., Atlas L.Y., Roy M., Wager T.D. (2020). Pain-evoked reorganization in functional brain networks. Cereb. Cortex.

[47] Androulakis X.M., Krebs K.A., Jenkins C., Maleki N., Finker A.G., Rorden C., Newman R. (2018). Central executive and default mode network intra-network functional connectivity patterns in chronic migraine. J. Neurol. Disord..

[48] Bishop J.H., Shpaner M., Kubicki A., Clements S., Watts R., Naylor M.R. (2018). Structural network differences in chronic muskuloskeletal pain: Beyond fractional anisotropy. NeuroImage.

[49] Seeley W.W. (2019). The salience network: A neural system for perceiving and responding to homeostatic demands. J. Neurosci..

