# Supplementary material for: Network Analysis of Induced Neural Plasticity Post-Acceptance and Commitment Therapy for Chronic Pain
Source: Brain Sci. 2020 Dec 23;11(1):10. doi: 10.3390/brainsci11010010 (PMC7823706; doi:10.3390/brainsci11010010)
Supplement: Supplementary file 1 [file brainsci-11-00010-s001.pdf]

Supplementary Material

Table S1. List of behavioral and neuropsychological assessments (administered pre- and post-treatment). *One of the assessments used in final correlational analysis (first pass) between behavioral data and rsfMRI functional connectivity data.

Battery/Assessment Name	Domain	Approximate Duration
NIH Toolbox Cognition Battery (iPad):		
List Sorting Working Memory (LSWM)	Working Memory	7 minutes
*Pattern Comparison Processing Speed Test (PCPS)	Processing Speed	3 minutes
Picture Sequence Memory Test (PSM)	Episodic Memory	7 minutes
Flanker Inhibitory Control and Attention Test (FIC)	Attention, Executive Function	3 minutes
Dimensional Change Card Sort Test (DCCS)	Executive Function	4 minutes
Auditory Verbal Learning Test (AVL)	Episodic Memory	3 minutes
Oral Symbol Digit Test (OSD)	Processing Speed	3 minutes
Patient-Reported Outcomes Measurement Information System (PROMIS; iPad):		
Pain Interference Survey	Pain	1 minute
Pain Intensity Survey	Pain	1 minute
Pain Behavior	Pain	7 items
Severity of Substance Use	Substance Abuse	7 items
Appeal of Substance Use	Substance Abuse	7 items
Prescription Pain Medication Misuse	Substance Abuse	7 items
Quality of Life in Neurological Disorders (Neuro QoL/NQ; iPad) - Short Form:		
*Satisfaction with Social Roles and Activities (SSR)	Quality of Life	25 minutes total:
Stigma	Quality of Life	
Positive Affect and Well-Being (PA)	Quality of Life	
*Fatigue	Quality of Life	
Emotional and Behavioral Dyscontrol (EBD)	Quality of Life	
*Depression	Quality of Life	
Anxiety	Quality of Life	
Ability to Participate in Social Roles and Activities (APSR)	Quality of Life	

Communication	Quality of Life	
Additional Assessments (paper):		
Current Opioid Misuse Measure (COMM)	Substance Abuse	17 items
*Center for Epidemiologic Studies Depression Scale (CESD)	Depression	20 items
*PTSD Checklist (PCL-5)	PTSD	20 items
Coping Strategies Questionnaire (CSQ)	Cognition	45 items
Barratt Impulsiveness Scale (BIS-11)	Impulsivity	30 items
*Brief Pain Inventory (BPI)	Pain	9 items
*Acceptance & Action Questionnaire (AAQ-II)	Pain	10 items
Chronic Pain Acceptance Questionnaire (CPAQ)	Pain	20 items
*Five Facet Mindfulness Questionnaire (FFMQ)	Mindfulness	39 items
Dissociative Experiences Scale - II (DES-II)	Cognition	28 items
	Approximate Total Time:	2 hours

Check in & set agenda	Review homework	Introduce the skill	Skill demonstration	In-session practice with feedback	Skill review	New homework

Figure S1. The seven-step timeline of each individual 90-minute ACT session. "Homework" involved continuing practice of the skill learned in the prior session, as well as completing self-monitoring and self-reporting activities.

Screening:<br><br>Eligibility, MRI screening	Pre-treatment scan and assessments:<br><br>Questionnaires for pain, cognitive measures, etc.<br><br>30-minute fMRI scan	ACT Treatment:<br><br>Two 90-minute sessions/week for four weeks	Post-treatment scan and assessments:<br><br>Questionnaires for pain, cognitive measures, etc.<br><br>30-minute fMRI scan

Figure S2. A basic timeline of the entire study, starting with pre-treatment screening and ending with post-treatment assessments.

Table S2. List of all X, Y, Z coordinates for the nodes involved in each of the four networks. The ROI labels are directly from the Power atlas, except for those of the pain network [29]. Pain nodes were derived from meta analytic connectivity modelling [31].

Default Mode Network				Frontoparietal Network			
**ROI**	**X**	**Y**	**Z**	**ROI**	**X**	**Y**	**Z**
**74**	-41	-75	26	**174**	-44	2	46
**75**	6	67	-4	**175**	48	25	27
**76**	8	48	-15	**176**	-47	11	23
**77**	-13	-40	1	**177**	-53	-49	43
**78**	-18	63	-9	**178**	-23	11	64
**79**	-46	-61	21	**179**	58	-53	-14
**80**	43	-72	28	**180**	24	45	-15
**81**	-44	12	-34	**181**	34	54	-13
**82**	46	16	-30	**186**	47	10	33
**83**	-68	-23	-16	**187**	-41	6	33
**86**	-44	-65	35	**188**	-42	38	21
**87**	-39	-75	44	**189**	38	43	15
**88**	-7	-55	27	**190**	49	-42	45
**89**	6	-59	35	**191**	-28	-58	48
**90**	-11	-56	16	**192**	44	-53	47
**91**	-3	-49	13	**193**	32	14	56
**92**	8	-48	31	**194**	37	-65	40
**93**	15	-63	26	**195**	-42	-55	45
**94**	-2	-37	44	**196**	40	18	40
**95**	11	-54	17	**197**	-34	55	4
**96**	52	-59	36	**198**	-42	45	-2
**97**	23	33	48	**199**	33	-53	44
**98**	-10	39	52	**200**	43	49	-2
**99**	-16	29	53	**201**	-42	25	30
**100**	-35	20	51	**202**	-3	26	44
**101**	22	39	39	Salience Network			
**102**	13	55	38	**ROI**	**X**	**Y**	**Z**
**103**	-10	55	39	**203**	11	-39	50
**104**	-20	45	39	**204**	55	-45	37
**105**	6	54	16	**205**	42	0	47
**106**	6	64	22	**206**	31	33	26
**107**	-7	51	-1	**207**	48	22	10
**108**	9	54	3	**208**	-35	20	0
**109**	-3	44	-9	**209**	36	22	3

**110**	8	42	-5
**111**	-11	45	8
**112**	-2	38	36
**113**	-3	42	16
**114**	-20	64	19
**115**	-8	48	23
**116**	65	-12	-19
**117**	-56	-13	-10
**118**	-58	-30	-4
**119**	65	-31	-9
**120**	-68	-41	-5
**121**	13	30	59
**122**	12	36	20
**123**	52	-2	-16
**124**	-26	-40	-8
**125**	27	-37	-13
**126**	-34	-38	-16
**127**	28	-77	-32
**128**	52	7	-30
**129**	-53	3	-27
**130**	47	-50	29
**131**	-49	-42	1
**137**	-46	31	-13
**139**	49	35	-12

**210**	37	32	-2
**211**	34	16	-8
**212**	-11	26	25
**213**	-1	15	44
**214**	-28	52	21
**215**	0	30	27
**216**	5	23	37
**217**	10	22	27
**218**	31	56	14
**219**	26	50	27
**220**	-39	51	17
Pain Network			
**ROI**	**X**	**Y**	**Z**
**1**	-36	18	2
**2**	-24	2	-2
**3**	40	-14	6
**4**	52	14	-2
**5**	4	16	44
**6**	58	-18	18
**7**	10	-4	2

Table S3. List of nodes presented in Figure 2 (A, B, C). The node assignment matches the 264 Power atlas nodes or 7 pain nodes found above.

Figure	Corresponding ROIs:	Network Association:
Figure 2(A)	94, 96, 119	Default Mode Network
	189, 192	Frontoparietal Network
	203, 204, 212, 215, 217	Salience Network
Figure 2(B)	94, 119	Default Mode Network
	192	Frontoparietal Network
	203, 212, 215	Salience Network
	2	Pain Network

Figure 2(C)	74, 76, 95, 110, 111, 112, 113, 115, 122, 130	Default Mode Network
	175, 189, 191, 194, 195, 196, 199, 202	Frontoparietal Network
	203, 204, 206, 208, 209, 211, 212, 215, 216	Salience Network
	1, 2, 4, 7	Pain Network

Supplement 1.1. Neuropsychological Testing

First, participants were asked to complete a series of neuropsychological assessments in their pre-treatment session to determine baseline measures of cognition, quality of life, and pain. Selected domains from the NIH Cognition Battery, PROMIS (Patient-Reported Outcomes Measurement Information System; regarding pain interference, pain intensity, etc.) and Neuro-QoL™ (Quality of Life in Neurological Disorders; regarding fatigue, depression, anxiety, etc.) were obtained from the NIH Toolbox and were administered via iPad. Any additional assessments were administered via paper copy. This set of assessments (Table S1) was administered to each subject both pre- and post-ACT.

Supplement 1.2. Neuropsychological Assessment Analysis

Assessment data were entered into Excel spreadsheets using Qualtrics software for statistical analysis. Analyses were conducted using SAS® v.9.4. to yield measures such as mean, standard deviation, Student's T (Change), Pr > |t|, Wilcoxon Signed Rank, and Pr >= |S| for a total score pre-ACT, total score post-ACT, and the change score between the two. Positive or negative change scores indicated satisfactory results, depending on the specific assessment in question (CESD scores decreasing meant less depression, and AAQ-II scores increasing meant greater feelings of acceptance, e.g.).

Supplement 1.3. Neuropsychological Assessment and rsfMRI Data Correlation Analysis

Additionally, the fMRI data set was merged with the neuropsychological assessment data set so that any correlations between change in neural plasticity (functional connectivity measures) and change in neuropsychological health indicators (behavioral measures) could be analyzed pre- and post-ACT. An Excel spreadsheet was compiled of each subject's pre- and post-ACT values for all significant edge connections (56 total; derived from an NBS analysis

between DMN, FPN, and SN with a threshold of t >2.1 instead of t >2.5; see Table S6) as well as certain significant change scores (9 reported; AAQ-II, BPI, CESD, FFMQ, PCL-5, Neuro-QoL Depression, Neuro-QoL Fatigue, Neuro-QoL Satisfaction with Social Roles and Activities, and NIH Toolbox Pattern Comparison Processing Speed; the CPAQ was not used). These 9 tests (which previously yielded significant change results; see Table S7) were selected for a more cohesive understanding of brain region activation as it corresponds to chronic pain-specific assessments. SAS® v.9.4 was again used to derive correlational data (pairwise Pearson correlation, R, and p value) between functional connectivity changes and neuropsychological assessment changes. For the first pass, all scores with a $p < 0.10$ were selected. The list was then cut down to seven assessment measures and 15 functional connectivity measures. For the second pass, all scores with a $p < 0.0071$ were selected (based on Bonferroni equation for multiple comparison correction), in addition to a $p < 0.05$.

Supplement 1.4. Neuropsychological Assessment Data Change Results

Improvements were found based on the assessment data from pre- to post-ACT. Of the administered tests, approximately half were found to have significant change scores (Table S7). Negative values for the following assessments represent: lower levels of pain severity (BPI), depression (CESD, Neuro-QoL Depression), PTSD (PCL-5), and fatigue (Neuro-QoL Fatigue). Positive values for the following assessments represent: higher levels of chronic pain acceptance and action (AAQ-II, CPAQ), mindfulness (FFMQ), processing speed (NIH Toolbox PCPS), and satisfaction with social roles (Neuro-QoL Satisfaction with Social Roles & Activities).

Supplement 1.5. Neuropsychological Assessment and rsfMRI Data Correlation Results

To further the investigation, the behavioral data and the resting state fMRI data were run together to search for any correlational relationships. Through multiple passes, the initial edges

and assessments were narrowed down to 15 edges and seven assessments. The final pass led to the correlations found in Table S8.

13 edges were included in significant ($p < 0.05$) correlations with six assessments (left column and top row in Table S8). The first assessment (AAQ) was correlated with one edge representing the functional connection between right AG and left ACC. The second assessment (BPI) was correlated with five edges representing the functional connections between left PHG and right IPL, right SFG and right PCG, right SFG and right IFG, right MFG and left ACC, and right SupMG and right ACC, respectively. The third assessment (FFMQ) was correlated with two edges representing the functional connections between left PHG and left PCG, and left PHG and right SFG, respectively. The fourth assessment (NQ DEP) was correlated with two edges representing the functional connections between right MFG and left ACC, and right PCG and right Ins, respectively. The fifth assessment (NQ SSR) was correlated with three edges representing the functional connections between right MTG and left PHG, left PHG and right SFG, and right IFG and right MFG, respectively. The sixth assessment (NIH Toolbox PCPS) was correlated with one edge representing the functional connection between left MCC and left ACC.

So, the significant changes in functional connectivity, namely within cingulate cortex and parahippocampal, precentral, and frontal gyri, were correlated with the significant changes in assessments regarding pain, social satisfaction, depression, and processing speed pre- to post-ACT. To correct for multiple comparisons, the data were assessed using a p value of 0.0071 (calculated by dividing 0.05 by the final number of assessments included, seven). Only one correlation involving the right angular gyrus and left anterior cingulate was significant using the Bonferroni method described (starred in Table S8).

Supplement 1.6. Main Findings in Neuropsychological Assessment Data

Behavioral data were collected using select domains for the NIH Cognition Battery, PROMIS and Neuro-QoL on the NIH Toolbox as well as physical paper surveys (Table S1). Approximately half of all of the assessments changed significantly. All of the significant change scores demonstrated improved scores for the nine participants (Table S7). All participants exhibited lower levels of pain severity, depression, PTSD, fatigue, as well as higher levels of chronic pain acceptance, mindfulness, processing speed, and satisfaction with their social roles. This indicates that the ACT enhanced quality of life in very important aspects of the overall perception of chronic pain condition. Chronic pain sufferers form negative life views more often than not. However, our study and previous studies point to ACT as a meaningful, non-invasive, life-enhancing treatment for various chronic pain conditions.

Previous studies of the behavioral outcomes post-ACT have found similar improvements for daily physical and social functioning in addition to alleviated psychological flexibility by improved depression and mindfulness scores. Others have demonstrated a resultant improvement of mental health measures of acceptance and value-based action, which would support the change score results on the AAQ-II in the current study. Dahl and Lundgren have demonstrated this acceptance to be linked to lower self-rated depression scores, greater physical and social abilities, and less pain avoidance, which would further support the change scores on the CESD, Neuro-QoL Depression, and Satisfaction with Social Roles assessments in the current study [23].

Supplement 1.7. Main Findings in Correlation Analysis Data

Because of the significant findings of the behavioral assessment analysis and the rsfMRI graph theory analysis, it was critical to then investigate if the two independent findings were correlated to each other. This additional correlational analysis was conducted (ultimately) using

seven behavioral assessment measures, 15 rsfMRI functional connectivity measures (edges), and the SAS® v.9.4 software. Pairwise Pearson correlations exhibited 14 significant correlations that were either strong (|0.6| – |0.8|) or very strong (> |0.8|) correlations (Table S8). Within the 14 correlations, four of the assessments (BPI, FFMQ, NQ DEP, and NQ SSR) correlated with more than one edge. This indicates important functional connections related to the improved assessment scores. Specifically, the BPI scores were significantly correlated with five connections (involving frontal gyri, cingulate, and more), demonstrating multiple important interactions between ACT-induced neural plasticity and subjective perception of pain severity.

Of importance is the connection between the right angular gyrus and the left anterior cingulate that was significantly correlated with the AAQ-II when correcting for multiple comparisons (using the Bonferroni method). At a p value of 0.0024 and R = 0.86811, this is a very strong correlation. The anterior cingulate has been previously linked to the cognitive and emotional regulation of pain processing as well as response selection, as opposed to aspects of pain intensity. In future investigations, the anterior cingulate and angular gyrus should be considered as potential underlying neural mechanisms. In addition, the behavioral measure for acceptance and action surrounding chronic pain should be studied more in depth.

Previous studies have also shown similar neurological interactions and revealed the importance of their connections. In numerous other ACT investigations with a chronic pain population, the medial frontal gyrus (found to have numerous significant correlations in the current study) has shown similar deactivation patterns. The medial frontal gyrus is namely responsible for assessing the risk of chronic pain as it manifests, possibly even before the chronicity is established [2]. When connections between the prefrontal cortex and anterior cingulate have been shown, those connections have been attributed to attentional and memory

network activation as a response to the painful stimuli. In discussing the anterior cingulate, it is important to note the region’s probable link to anxiety and depression (as depression measures were decreased in the current study). Additional studies have also demonstrated activation changes in the anterior cingulate after ACT interventions, further indicating its level of interaction.

Table S4. List of (three) significant edges of the salience network involving four nodes. The node assignment is listed out of the 264 Power atlas nodes. The brain regions corresponding to the nodes of each connection are listed also.

Power ROI	Corresponding Brain Regions:
203, 212	Right medial cingulate cortex (MCC), Left anterior cingulate cortex (ACC)
204, 212	Right supramarginal gyrus (SupMG), Left anterior cingulate cortex (ACC)
204, 217	Right supramarginal gyrus (SupMG), Right anterior cingulate cortex (ACC)

Table S5. List of the (34) significant edges (abbreviations) of triple network (t >3.4) found in Figure 6 with corresponding brain regions. The node assignment is listed out of the 264 Power atlas nodes. Items listed in **bold** are from the pain ALE [35].

ROI List	Abbreviation	Corresponding Brain Regions
3, 35	R MFG, R MFG	R medial frontal gyrus, R medial frontal gyrus
36, 40	L ACC, L SFG	L anterior cingulate, L superior frontal gyrus
20, 55	R Pre, R Ang	R precuneus, R angular gyrus
36, 60	L ACC, R IFG	L anterior cingulate, R inferior frontal gyrus
47, 70	R ACC, R MFG	R anterior cingulate, R medial frontal gyrus
1, 72	L MOcc, L SPL	L medial occipital lobe, L superior parietal lobule
3, 75	R MFG, R Ang	R medial frontal gyrus, R angular gyrus
55, 75	R Ang, R Ang	R angular gyrus, R angular gyrus
1, 76	L MOcc, L IPL	L medial occipital lobe, L inferior parietal lobule
3, 77	R MFG, R MFG	R medial frontal gyrus, R medial frontal gyrus
36, 80	L ACC, R IPL	L anterior cingulate, R inferior parietal lobule
37, 80	L SFG, R IPL	L superior frontal gyrus, R inferior parietal lobule
38, 80	L ACC, R IPL	L anterior cingulate, R inferior parietal lobule
40, 80	L SFG, R IPL	L superior frontal gyrus, R inferior parietal lobule
1, 83	L MOcc, L SFG	L medial occipital lobe, L superior frontal gyrus
38, 84	L ACC, R MCC	L anterior cingulate, R medial cingulate
3, 85	R MFG, R SMG	R medial frontal gyrus, R supramarginal gyrus
37, 85	L SFG, R SMG	L superior frontal gyrus, R supramarginal gyrus
47, 90	R ACC, R Ins	R anterior cingulate, R insula
87, 90	R MFG, R Ins	R medial frontal gyrus, R insula
47, 92	R ACC, R Ins	R anterior cingulate, R insula
90, 92	R Ins, R Ins	R insula, R insula
80, 93	R IPL, L ACC	R inferior parietal lobule, L anterior cingulate
1, 96	L MOcc, L ACC	L medial occipital lobe, L anterior cingulate

80, 96	R IPL, L ACC	R inferior parietal lobule, L anterior cingulate
89, 96	L Ins, L ACC	L insula, L anterior cingulate
96, **102**	L ACC, **L Ins**	L anterior cingulate, **L insula**
97, **102**	R MCC, **L Ins**	R medial cingulate, **L insula**
75, **103**	R Ang, **L Put**	R angular gyrus, **L putamen**
**102, 105**	**L Put, R Ins**	**L putamen, R insula**
87, **108**	R MFG, **R Thal**	R medial frontal gyrus, **R thalamus**
89, **108**	L Ins, **R Thal**	L insula, **R thalamus**
90, **108**	R Ins, **R Thal**	R insula, **R thalamus**
**102, 108**	**L Ins, R Thal**	**L insula, R thalamus**

Table S6. List of (56) significant edges of the triple network (t >2.1). The node assignment is listed out of the 264 Power atlas nodes. The brain regions corresponding to the nodes of each connection are listed also, with the abbreviations used in this paper.

ROI List	Corresponding Power Atlas Brain Regions
96, 104	Right angular gyrus (AG), Left superior frontal gyrus (SFG)
76, 124	Right medial frontal gyrus (orbital, MFG), Left parahippocampal gyrus (PHG)
111, 124	Left anterior cingulate cortex (ACC), Left parahippocampal gyrus (PHG)
116, 124	Right medial temporal gyrus (MTG), Left parahippocampal gyrus (PHG)
123, 124	Right medial temporal gyrus (MTG), Left parahippocampal gyrus (PHG)
76, 126	Right medial frontal gyrus (orbital, MFG), Left fusiform gyrus (FG)
80, 126	Right medial occipital gyrus (MOG), Left fusiform gyrus (FG)
123, 126	Right medial temporal gyrus (MTG), Left fusiform gyrus (FG)
96, 127	Right angular gyrus (AG), Right cerebellum (Cer)
124, 174	Left parahippocampal gyrus (PHG), Left precentral gyrus (PCG)
124, 175	Left parahippocampal gyrus (PHG), Right inferior frontal gyrus (triangular, IFG)
124, 176	Left parahippocampal gyrus (PHG), Left inferior frontal gyrus (opercular, IFG)
126, 176	Left fusiform gyrus (FG), Left inferior frontal gyrus (opercular, IFG)
124, 178	Left parahippocampal gyrus (PHG), Left superior frontal gyrus (SFG)
126, 178	Left fusiform gyrus (FG), Left superior frontal gyrus (SFG)
124, 180	Left parahippocampal gyrus (PHG), Right superior frontal gyrus (orbital, SFG)

126, 180	Left fusiform gyrus (FG), Right superior frontal gyrus (orbital, SFG)
175, 180	Right inferior frontal gyrus (triangular, IFG), Right superior frontal gyrus (orbital, SFG)
139, 181	Right inferior frontal gyrus (orbital, IFG), Right medial frontal gyrus (orbital, MFG)
124, 188	Left parahippocampal gyrus (PHG), Left medial frontal gyrus (MFG)
126, 188	Left fusiform gyrus (FG), Left medial frontal gyrus (MFG)
180, 189	Right superior frontal gyrus (orbital, SFG), Right medial frontal gyrus (MFG)
124, 191	Left parahippocampal gyrus (PHG), Left superior parietal gyrus (SPG)
124, 192	Left parahippocampal gyrus (PHG), Right inferior parietal lobule (IPL)
126, 192	Left fusiform gyrus (FG), Right inferior parietal lobule (IPL)
188, 194	Left medial frontal gyrus (MFG), Right angular gyrus (AG)
124, 199	Left parahippocampal gyrus (PHG), Right inferior parietal lobule (IPL)
124, 201	Left parahippocampal gyrus (PHG), Left inferior frontal gyrus (triangular, IFG)
126, 201	Left fusiform gyrus (FG), Left inferior frontal gyrus (triangular, IFG)
180, 205	Right superior frontal gyrus (orbital, SFG), Right precentral gyrus (PCG)
124, 207	Left parahippocampal gyrus (PHG), Right inferior frontal gyrus (triangular, IFG)
180, 207	Right superior frontal gyrus (orbital, SFG), Right inferior frontal gyrus (triangular, IFG)
180, 208	Right superior frontal gyrus (orbital, SFG), Left insula (Ins)
124, 209	Left parahippocampal gyrus (PHG), Right insula (Ins)
205, 209	Right precentral gyrus (PCG), Right insula (Ins)
124, 210	Left parahippocampal gyrus (PHG), Right inferior frontal gyrus (orbital, IFG)
126, 210	Left fusiform gyrus (FG), Right inferior frontal gyrus (orbital, IFG)
94, 212	Left medial cingulate cortex (MCC), Left anterior cingulate cortex (ACC)
96, 212	Right angular gyrus (AG), Left anterior cingulate cortex (ACC)
119, 212	Right medial temporal gyrus (MTG), Left anterior cingulate cortex (ACC)
124, 212	Left parahippocampal gyrus (PHG), Left anterior cingulate cortex (ACC)
126, 212	Left fusiform gyrus (FG), Left anterior cingulate cortex (ACC)
189, 212	Right medial frontal gyrus (MFG), Left anterior cingulate cortex (ACC)
192, 212	Right inferior parietal lobule (IPL), Left anterior cingulate cortex (ACC)

203, 212	Right medial cingulate cortex (MCC), Left anterior cingulate cortex (ACC)
204, 212	Right supramarginal gyrus (SupMG), Left anterior cingulate cortex (ACC)
126, 214	Left fusiform gyrus (FG), Left medial frontal gyrus (MFG)
203, 214	Right medial cingulate cortex (MCC), Left medial frontal gyrus (MFG)
181, 215	Right medial frontal gyrus (orbital, MFG), Left anterior cingulate cortex (ACC)
189, 215	Right medial frontal gyrus (MFG), Left anterior cingulate cortex (ACC)
192, 215	Right inferior parietal lobule (IPL), Left anterior cingulate cortex (ACC)
196, 215	Right medial frontal gyrus (MFG), Left anterior cingulate cortex (ACC)
124, 217	Left parahippocampal gyrus (PHG), Right anterior cingulate cortex (ACC)
189, 217	Right medial frontal gyrus (MFG), Right anterior cingulate cortex (ACC)
204, 217	Right supramarginal gyrus (SupMG), Right anterior cingulate cortex (ACC)
215, 218	Left anterior cingulate cortex (ACC), Right superior frontal gyrus (SFG)

Table S7. Statistically significant change scores of assessments administered.

Neuropsychological Assessment (Change Score)	S	Pr >= |S|	T	Pr > |t|
AAQ-II	20	0.0156		
BPI	-16.5	0.0234		
CESD	-18.5	0.0273		
CPAQ	20	0.0156		
FFMQ	20.5	0.0117		
PCL-5	-21.5	0.0078		
Neuro-QoL Depression	-17.5	0.0352	-2.37633	0.0448
Neuro-QoL Fatigue	-17	0.0156	-3.16337	0.0133
Neuro-QoL Satisfaction with Social Roles & Activities	22.5	0.0039	2.35737	0.0461
NIH Toolbox Pattern Comparison Processing Speed (PCPS) Test:	-	-	-	-
Age Corrected Standard Score	22.5	0.0039	7.49688	<0.0001
Computed Score	22.5	0.0039	6.11596	0.0003
Fully Corrected Score	22.5	0.0039	8.176373	<0.0001

Item Count	22.5	0.0039	5.962922	0.0003
National Percentile (Age Adjusted) Score	22.5	0.0039	4.625803	0.0017
Uncorrected Standard Score	22.5	0.0039	6.189544	0.0003

Note. S: Wilcoxon Signed Rank; T: Student's T (Change). T scores were not reported for assessments administered by paper (AAQ-II, e.g.) because normality could not be assumed. Change scores represent the difference in score between the first and second timepoints (negative S and T values indicate that the score post-ACT was lower than pre-ACT, while positive S and T values indicate post-ACT scores higher than pre-ACT).

Table S8. Correlations between significant assessment and edge scores.

	AAQ	BPI	FFMQ	NQ_DEP	NQ_SSR	PCPS
**L MCC, L ACC**						0.77662 **0.0138**
**R AG, L ACC**	0.86811 **0.0024***					
**R MTG, L PHG**					-0.74181 **0.0221**	
**L PHG, L PCG**			-0.81066 **0.008**			
**L PHG, R SFG**			-0.68596 **0.0413**		-0.81151 **0.0079**	
**L PHG, R IPL**		-0.77014 **0.0152**				
**R IFG, R MFG**					-0.6976 **0.0367**	
**R SFG, R PCG**		-0.7447 **0.0213**				
**R SFG, R IFG**		-0.69481 **0.0378**				
**R MFG, L ACC**				-0.66811 **0.0492**		
**R MFG, L ACC**		-0.76687 **0.0159**				
**R SupMG, R ACC**		-0.79017 **0.0113**				
**R PCG, R Ins**				0.67389 **0.0466**		

Note. All significant correlations between edge and assessment change scores. P values are bold, listed below the R values. *This represents the only value (0.0024) that is < 0.0071 of the significant correlations.
